# Editorial

**Published:** 1839-02-09

**Authors:** 


### THE MEDICAL EXAMINER.

PHILADELPHIA, FEB. 9, 1839.

In our last number, we insisted on the importance of an accurate examination of the ovaria in determining the question of recent parturition, where the female had died. We stated our conviction, that the detection of a genuine corpus luteum would furnish unequivocal proof, other circumstances concurring, of delivery having taken place within a short period. Our readers are doubtless aware that some attempts have been made to discredit the value of this sign; but the discordant testimony of physiologists has arisen, we are inclined to believe, from the imperfect and erroneous views which have been entertained respecting the real nature of this structure. The industry and ability of one individual, within a few years, have divested the subject of all obscurity, and rendered it singularly clear and lucid. We need hardly add, that we allude to Prof. Montgomery, of Dublin. Of the invaluable labours of this gentleman we shall liberally avail ourselves, in the course of this article. Of such paramount importance do we deem a correct knowledge of this evidence of conception, that we propose here to devote some space in attempting to determine its precise value. To effect this, it will be necessary for us to state, at the outset, what we understand by a corpus luteum. An appreciable difference exists in the ovaria of a recently impregnated woman. On the one from which the germ has been derived, a tumour is seen, having the appearance of a "segment of a smaller circle, superimposed on that of a greater," marked on its surface by a cicatrix, and differing in colour from the remainder of the organ. Upon making a vertical section, we expose an oval, vascular, glandular structure, about four-eighths of an inch in its long diameter, and of a dull yellow colour. If the examination be made within four or five months after conception, a cavity will be found in its centre, of sufficient capacity to contain a grain of wheat.* The sides of this cyst gradually approximate, until finally adhesion occurs, and we have in its place a *radiated white line.* So long as there exists any trace of the corpus luteum, this is to be found, and is the distinctive, essential character of this body, distinguishing it from every other with which it might be confounded. On the cessation of gestation, whether by delivery at term, or the premature expulsion of the ovum, an alteration commences in the corpus luteum, and at the end of five or six months no reliable trace of it is to be found. Montgomery says, that "the corpus luteum of a preceding conception is never to be found along with that of a more recent, when gestation has arrived at its full term; but in cases of miscarriage, repeated at short intervals, it may." The corpora lutea correspond exactly in all cases with the number of vivified ova; and where more than one exist in the ovary, and but one fœtus is found in the womb, diligent search invariably detects blighted ova either in the uterus or the tubes.

Malpighi, Bertrandi, Santorini, and some other of the old anatomists, believed that the corpus luteum was a mere provision for conception, within which the ovum is prepared for impregnation. This doctrine was revived in 1809, by Sir Everard Home,† who claimed its paternity. His hypothesis is founded on a solitary case, and no explanation or argument is attempted in its support. It rests upon mere assertion. If this view were correct, corpora lutea ought to be detected in every woman who has arrived at puberty, and so long as there is any aptitude for procreation,—and their size should be in a direct inverse ratio to the last period of conception. The reverse of these conditions, however, obtains,—and their number, as we have stated, corresponds with the number of successful impregnations. Moreover, the cicatrix on the surface cannot be accounted for in this way. Another assertion, totally inconsistent with the one we have just controverted, and rendering the value of this sign utterly nugatory, is, that corpora lutea may result from strong venereal excitement. Were this correct, we should invariably find them in all cases where the female had been exposed to a high degree of sexual excitation. But experience proves incontrovertibly the reverse. "Of the non-occurrence of which consequence," says Prof. Montgomery, "we can speak in very decided terms, from numerous opportunities of making examinations under such circumstances." Here is positive evidence opposed to mere assertion, or visionary theory.

We shall now quote the opinions of some of the most celebrated physiologists, in support of the view we have taken.

Haller, who was fully aware of the existence of an opposite opinion, and, after years of careful and extended investigation, affirmed, "nullus unquam conceptus est absque corpore luteo," and that "corpus luteum in virgineis animalibus nullum est, ex conceptione oritur." In 1797, Dr. Haighton declared, after innumerable experiments on the subject of animal impregnation, "that no corpora lutea exist in virgin animals, and that whenever they are found, they furnish incontestible proof that impregnation either does exist or has preceded." Drs. William Hunter, Blundell, and Baillie, entertained the same views. De Graaf holds this language:—"Hos globulos non omni tempore in fœmellarum existere dicimus; quia *post coitum tantum* in illis detegentur, unus aut plures, prout animal ex illo congressu unum aut plures fœtus in lucem

* According to the celebrated physiologist, Prof. Von Baer, the corpus luteum is nothing more than a mucous membrane lining the Graffian vesicle, which becomes detached on the escape of the vivified ovum, as proved by its corrugation on cutting through it. Besides a long and successful devotion to embryology, Prof. Von B. enjoyed the rare opportunity of examining a female who committed suicide eight days subsequent to impregnation.

† The merited obloquy which now covers the private character of Sir Everard Home, ought not to be extended to his professional reputation, unless there exist undoubted proof of its being equally deserved. One who knew him well, holds this language concerning him:—"But we think he is yet too highly estimated as a man of science; and we know in conversation at meetings professedly scientific, his sentiments and language were so gross, as to justify a belief that his attainments were as limited as his mind was impure. We allude to this circumstance, to warn students of youthful age against the worship of such unclean deities, whose temporary influence is often great, but never salutary." So notorious an example of dishonesty, treachery, and ingratitude, rarely, we suspect, if ever, before disgraced the scientific annals of any country.

edit." Mr. Cruikshank speaks of them as "a certain mark of conception in all quadrupeds, and in women themselves, whether the embryo is visible or not." Dr. Denman, Messrs. H. Cline and Abernethy, and Sirs Charles M. Clarke and Astley Cooper, certified in the Angus case to the same effect. Dr. Seymour says that he has "examined the ovaria in the human being, and in animals, at the period of puberty, in very many instances; many had ova ready for impregnation, large, projecting, vascular, yet no corpora lutea were visible." Meckel has been cited in opposition to this doctine. We shall quote his own words. "In fact, several rare cases, where yellow bodies have been found in unmarried females and virgins, and always attended with the phenomena mentioned above, lead us to think that the formation of these bodies had been *preceded by the act of coition, and by impregnation.*"* We shall now state Professor Montgomery's opinion, the result of ten years of diligent observation, unwearied research, and careful reflection. "We never, in any one instance, saw the corpus luteum, having the characters we have described as belonging to it, except in females who had previously been impregnated and had conceived, and our firm conviction is, that such a corpus luteum was never found in a virgin animal." "We have dissected hundreds of the inferior animals with reference to this point, and have in our museum preparations of ovaries, exhibiting the corpus luteum in the human female, and also in cows, mares, sheep, sows, goats, bitches, cats, hares, rabbits; and our firm conviction is of the truth of both of Haller's propositions, viz.: That '*conception never happens without the production of a corpus luteum*,' and that '*the corpus luteum is never found in virgin females, but is the effect of impregnation.*' Again—'The value of which [corpus luteum] has been already so fully considered, that it appears now only necessary to remark, that although its existence is *proof positive of conception*,' &c. &c."

The difference of opinion on this point has arisen from several causes. Many individuals have been contented with a simple iteration of the assertion of others, and have been at no pains to investigate the subject and satisfy themselves, and in many cases have grossly misrepresented and perverted the very authorities they quote. The name of Blumenbach has been invoked by those who maintain the existence of corpora lutea independent of previous impregnation. No where in his writings does he positively assert this doctrine, but merely states his *suspicion* that *others* who have embraced and advocated it, are correct in their views. This belief he distinctly avers, is not the result of actual observation, but is entertained merely from the circumstance that certain writers had detected corpora lutea in the ovaria of some Italian girls, and he conjectures that the climate may have had some influence in their production.

Not unfrequently, singular ignorance of the true nature of a corpus luteum has been displayed by authors, some confounding the external cicatrix with it,* and others supposing that every point of yellow or dusky hue in the ovaria, are corpora lutea, forgetting Meckel's assertion, that "every yellow substance found in the ovary is not a yellow body."† Dr. Dunlop, the English commentator of Beck, in attempting to invalidate this evidence of pregnancy, triumphantly declares, that he found in the ovaria of a girl not five years old, "numerous corpora lutea as distinct as he ever saw them in the adult impregnated female." Dr. Montgomery rebukes this reckless assertion in these words, "one *real* corpus luteum, as it is found in the 'adult impregnated female,' is fully as large, or even larger, than the ovary of a child of five years."

Dr. Montgomery's description of these pseudo, or virgin copora lutea, as they are sometimes improperly called, is so graphic and clear, that we shall extract it entire.

"1. There is no prominence or enlargement of the ovary over them. 2. The external cicatrix is almost always wanting. 3. They are often, several of them, in both ovaries, especially in patients who have died of phthisis, when they appear to be tubercular depositions, without any discoverable connexion with the Graffian vesicles. 4. They are not vascular and cannot be injected. 5. Their texture is sometimes so infirm that they seem to consist merely of the remains of a coagulum, and at others appear fibro-cellular, and resembling

* Manual of General, Descriptive, and Pathological Anatomy, by J. F. Meckel, Doane's Translation, volume 3, page 492.

* The cicatrices found in the ovum are not only frequently mistaken for the corpora lutea, but are commonly supposed to be permanent. This is erroneous. Women may have borne many children, yet only one or more cicatrices be found. Professor Montgomery examined a female, the mother of eight children, and one cicatrix only was found in each ovary. Cicatrices caused by the rupture of small abscesses, cannot be distinguished from those which are produced by the escape of a vivified germ.

† Meckel, op. cit., vol. 3., p. 439.

that of the internal structure of the ovary, but in no instance did we ever see them presenting the soft, rich, and regularly glandular appearance which Hunter meant to express, when he described them as 'tender and friable, like glandular flesh.' 6. In form they are often triangular and square, or of some figure bounded by straight lines. 7. They never present either the central cavity, or the radiated or stelliform, which results from its closure."

From what has been said, the reader will, we feel certain, concur with us in the belief, that though the presence of a corpus luteum in either ovary, is not positive evidence of a woman having *borne* a child, it will, if found in conjunction with the commonly recognised signs of recent delivery, warrant the unequivocal juridical opinion that a fœtus has been expelled from the womb, within a short period.

