# Erratum in No. 6

**Published:** 1839-02-16

**Authors:** 


### ERRATUM IN NO. 6.

In the foot note at page 96, first line, for *ovum*, read *ovaria*.

